# Temperature Effect on Capillary Flow Dynamics in 1D Array of Open Nanotextured Microchannels Produced by Femtosecond Laser on Silicon

**DOI:** 10.3390/nano10040796

**Published:** 2020-04-21

**Authors:** Ranran Fang, Hongbo Zhu, Zekai Li, Xiaohui Zhu, Xianhang Zhang, Zhiyu Huang, Ke Li, Wensheng Yan, Yi Huang, Valeriy S. Maisotsenko, Anatoliy Y. Vorobyev

**Affiliations:** 1 School of Science, Chongqing University of Posts and Telecommunications, 2 Chongwen Road, Nanan District, Chongqing 400065, China; fangrr@cqupt.edu.cn (R.F.); huangzhiyu1026@hotmail.com (Z.H.); kerry_hhhh@163.com (K.L.); 2 School of Photoelectrical Engineering, Chongqing University of Posts and Telecommunications, 2 Chongwen Road, Nanan District, Chongqing 400065, China; LZK_KAI@163.com (Z.L.); zxh15111976635@163.com (X.Z.); 18323656339@163.com (X.Z.); yws118@gmail.com (W.Y.); huangy@cqupt.edu.cn (Y.H.); 3 M-Cycle Corporation, 1120 Delaware St. #110, Denver, CO 80204, USA; valeriymaisotsenko@gmail.com

**Keywords:** femtosecond laser processing, nanostructures, microstructures, open capillary microchannels, capillary flow, silicon, cooling of electronics, Maisotsenko cycle

## Abstract

Capillary flow of water in an array of open nanotextured microgrooves fabricated by femtosecond laser processing of silicon is studied as a function of temperature using high-speed video recording. In a temperature range of 23–80 °C, the produced wicking material provides extremely fast liquid flow with a maximum velocity of 37 cm/s in the initial spreading stage prior to visco-inertial regime. The capillary performance of the material enhances with increasing temperature in the inertial, visco-inertial, and partially in Washburn flow regimes. The classic universal Washburn’s regime is observed at all studied temperatures, giving the evidence of its universality at high temperatures as well. The obtained results are of great significance for creating capillary materials for applications in cooling of electronics, energy harvesting, enhancing the critical heat flux of industrial boilers, and Maisotsenko cycle technologies.

## 1. Introduction

Cooling of high-heat flux electronics is a long-standing problem [1,2,3] that has become a critical one with the advent of 5G networks and associated 5G electronic devices. Heat removal through the liquid–vapor phase transition is the most efficient approach to dissipate high heat fluxes because of high latent heat of the liquid–vapor phase transition. A number of cooling devices have been developed using this approach, such as heat pipes [4,5,6], spray cooling [7,8,9,10], and microfluidic heat sinks [11]. Because of simplicity, heat pipes are widely used in cooling electronics. They transfer heat by evaporating and condensing the working liquid (typically de-ionized water [5]) inside an evacuated and hermetically sealed enclosure. The vapor produced in a heat pipe evaporator rapidly spreads to a condenser, where it is cooled and converted to the liquid that flows back to the evaporator because of a wicking material between the condenser and evaporator [5]. Currently, a common wicking material is a sintered metal powder that creates a relatively high fluid pumping pressure, providing a cooling heat flux up to 140 W/cm^2^. However, the flow of liquid in the porous wicks is slow that limits their application at high heat fluxes. The recent advent of 5G electronic devices with increased heat flux has generated the demand for more efficient wicking materials for enhancing the performance of heat pipes and other two-phase cooling devices. A critical problem in cooling of advanced integrated circuits is the formation of dry-out spots caused by both localized hot spots that can generate heat fluxes as high as 1000–1500 W/cm^2^ [1,2] and non-uniform wetting of a hot surface in phase-change cooling devices. This problem necessitates the creation of wicking materials with a high velocity of capillary flow for quick rewetting of dry-out spots. 

Recently, novel wicking materials have been created using femtosecond laser nano/microstructuring technology [12,13,14,15] based on the direct laser ablation [16,17,18,19,20]. It has been demonstrated that 1D array of open nanotextured microchannels produced on a silicon surface provides fast capillary flow of water over a long distance even against gravity [21]. This strong wicking effect comes from a unique hierarchical pattern of surface structures formed by femtosecond laser processing. The created hierarchical surface structure contains microgrooves (80–100 microns across) covered with random fine microstructures and nanostructures. The velocity of capillary flow in this hierarchical wicking structure reaches several centimeters per second at room temperature, indicating its promising potential for applications in two-phase cooling of electronics. Here, we apply this femtosecond laser technology to produce superwicking silicon and perform a detailed study of its capillary properties at various temperatures to assess its suitability for both liquid-vapor phase-change cooling of electronics and other potential applications.

Despite many researches on evaporation processes of a liquid on a hot wicking surface [22,23,24,25,26,27,28,29,30,31,32,33], the spreading dynamics of a liquid in a heated wicking surface structure under conditions of evaporation remains a poorly studied issue [34,35,36,37,38]. Here, we investigate the capillary flow dynamics in an open capillary surface structure at temperatures between 23 and 80 °C. Previous studies show that the capillary flow velocity has its maximum in the beginning the liquid spreading [39]. Therefore, understanding of the capillary flow in its early stages is critically important in solving the problem of quick rewetting of dry-out spots, and we perform a detail study of the capillary dynamics in these stages. Using high-speed video recording, we find that the maximum water flow velocity in our wicking material achieves an extremely high value of 37 cm/s in the capillary flow stages preceding the classic Washburn regime. The very strong capillary action of the studied silicon material provides water transport for a distance of 10 mm in only 40 ms from the beginning of capillary flow, demonstrating its ability of quick rewetting of dry-out spots. In this work, our important finding is the effect of enhancing the capillary action with increasing temperature. The obtained results show a high potential of the created wicking material for cooling applications and provide the guidelines for the creation of other high-temperature wicking materials, including metals, ceramics, and polymers needed in such applications as Maisotsenko cycle (M-cycle) technologies [40,41], water steam injection systems for reducing both fuel consumption and NO*x* emissions of internal combustion engines [42,43], energy harvesting [44,45,46], enhancing the critical heat flux of industrial boilers [47], evaporation-driven engines [48], capillary micromolding [49,50], printed electronics [51], and capillary coatings [50].

## 2. Experimental: Fabrication and Characterization 

**Figure 1:**
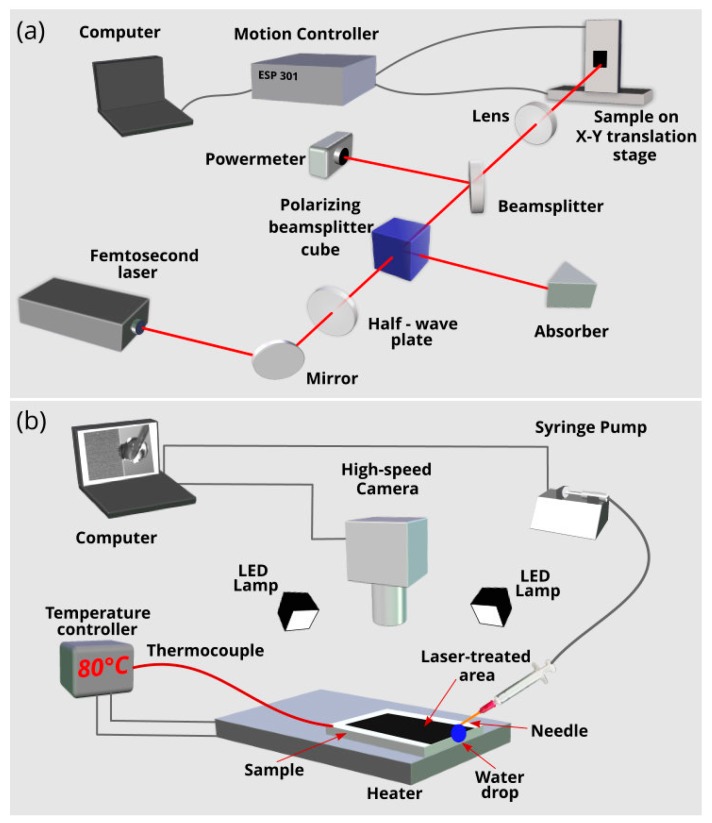
(**a**) Femtosecond laser setup for fabrication of the array of parallel microgrooves. (**b**) Experimental setup for high-speed video recording of capillary flow dynamics at various temperatures.

In our study, we use single-crystal phosphorus-doped silicon [(100)-oriented, resistivity of 1–30 Ω·cm]. The laser setup for fabrication of surface wicking nano/microstructures is shown in Figure 1a. A femtosecond laser (Astrella, Coherent Inc., Santa Clara, CA, USA) generates 86-fs pulses with energy around 7.2 mJ/pulse at a maximum repetition rate of 1 kHz with a central wavelength of 800 nm. The horizontally polarized laser beam is focused by a lens onto silicon sample mounted on a computer-controlled XY translation stage from Newport Corporation (Irvine, CA, USA). The laser power is varied using a half-wave plate and polarizing beamsplitter cube. A non-polarizing beamsplitter and powermeter are used for measuring the laser power. An array of parallel microgrooves is produced by a raster scanning the sample across the laser beam. To find laser processing parameters for producing a highly efficient wicking structure, we vary laser fluence, scanning speed, step between scanning lines, and pulse repetition rate. Laser processing of the sample is performed in air of atmospheric pressure. In this study, the wicking surface structure is produced using laser fluence of 6.3 J/cm^2^, pulse repetition rate of 1000 Hz, step between scanning lines of 100 µm, and scanning speed of 1 mm/s. The size of the laser-treated area is 15 mm × 45 mm. The morphology of the produced wicking structures is characterized by using a 3D laser scanning microscope VK-X1100 from Keyence and scanning electron microscope (SEM) Sigma 300 from Zeiss (Jena, Germany). Elemental composition of both the treated and untreated sample surfaces is examined by an energy dispersive X-ray spectroscopy (EDS) using a Brucker XFlash 6/30 detector (Karlsruhe, Germany). 

The capillary flow dynamics of a liquid (de-ionized water) is studied on a horizontally-positioned sample using the experimental setup shown in Figure 1b. The studied sample is mounted on a heater. The sample temperature is measured with a thermocouple. A syringe pump Elite 11 from Harward Apparatus Inc. (Holliston, MA, USA) is used for producing 10 µL pendant droplets. A pendant droplet on the tip of a syringe needle is brought in a contact with the studied wicking structure by translating the sample horizontally. The moment of the contact (*t* = 0) can be clearly identified by deformation of the droplet shape. The studied liquid is de-ionized water at room temperature of 23 °C. A high-speed VEO 710L Phantom camera at a speed of 1000 frames per second (fps) is used to capture liquid spreading. The spatial resolution of video recording is about 50 µm. The water spreading distance *h* is measured with high-speed camera software. To clearly see the propagating water front, the video frames are enlarged by 4–6×. The temperature effects on capillary flow dynamics are studied at *T* = 23, 60, and 80 °C. All experiments are conducted in air at a fixed relative humidity of 50%. Reproducibility of the capillary flow was tested by capturing three videos for each studied temperature.

## 3. Results and Discussion

**Figure 2:**
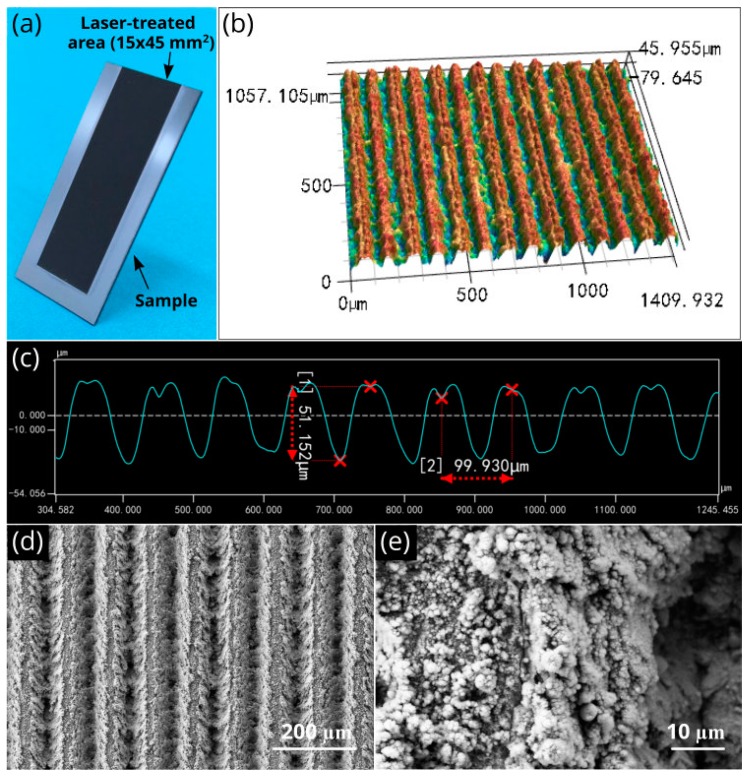
(**a**) Photograph of the wicking silicon sample. (**b**) 3D optical image of the array of parallel microgrooves. (**c**) Microgroove profile. (**d**) Scanning electron microscope (SEM) image of parallel microgrooves. (**e**) Nano- and micro-structural features of a microgroove.

A photograph of the silicon sample after femtosecond laser processing is demonstrated in Figure 2a, where the laser-treated area appears black because of the modification of the optical properties caused by surface texturing [12]. Morphology of the produced 1D array of parallel microgrooves is shown in Figure 2b–e. As seen in Figure 2c, the period and depth of the microgrooves are about 100 and 51 µm, respectively. SEM study (see Figure 2d,e) shows that the surface of microgrooves is textured with irregular nano- and fine micro-structures of various shapes. The size of these irregular structures is in a range between 45 nm and 5 µm. Thus, the produced wicking structure is a hierarchical one with structural features in a range between 45 nm and 100 µm. EDS analysis reveals a small increase in the amount of oxygen on the laser-treated area because of laser-induced oxidation. 

**Figure 3:**
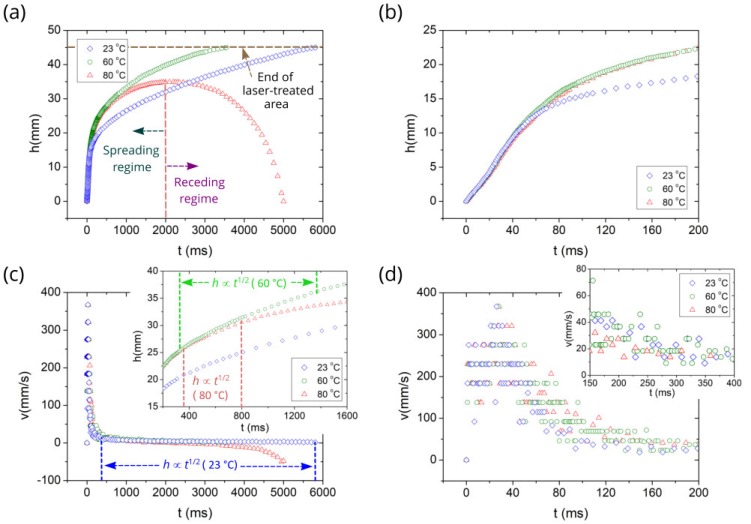
(**a**) Plot of the spreading distance as a function of time at various temperatures. (**b**) Detailed plot of the initial stage taken from (**a**). (**c**) Plot of spreading front velocity as a function of time at various temperatures. The inset shows the Washburn regime timescale at 60 and 80 °C. (**d**) Detailed plot of the velocity in the initial stage. The inset shows the plot of velocity as a function of time between 150 and 400 ms.

**Figure 4:**
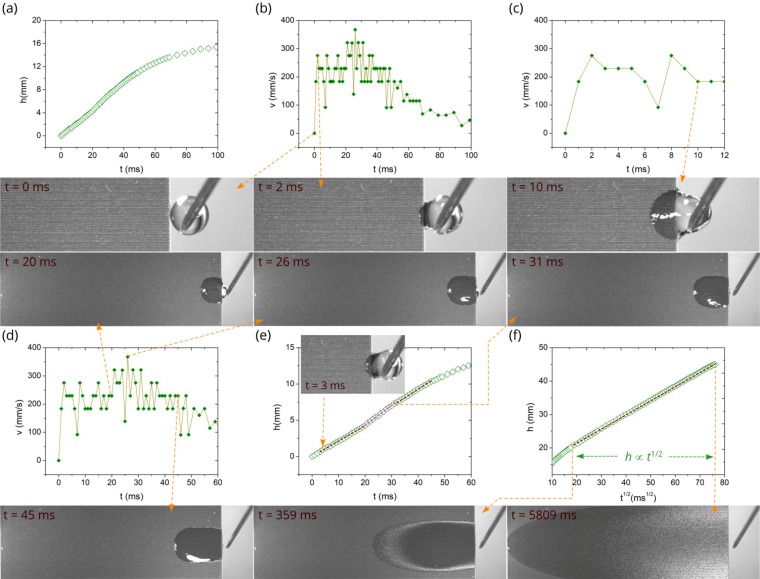
Detailed *h*(*t*) and *v*(*t*) plots along with snapshots of water spreading at room temperature. (**a**) Plot of the spreading distance as a function of time between 0 and 100 ms. (**b**) Plot of the velocity as a function of time. (**c**) Plot of the velocity as a function of time between 0 and 12 ms. (**d**) Plot of the velocity as a function of time between 0 and 60 ms. (**e**) Detailed plot of spreading distance as a function of time between 0 and 60 ms. (**f**) Plot of the spreading distance as a function of *t*^1/2^.

**Figure 5:**
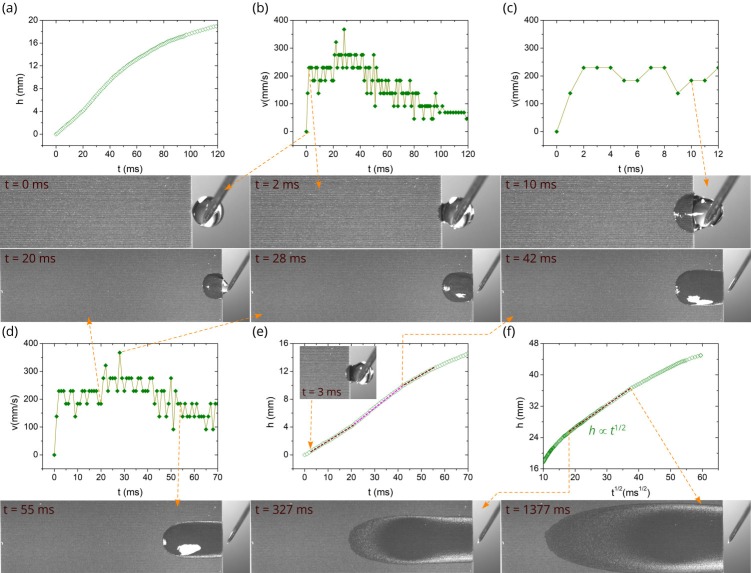
Detailed *h*(*t*) and *v*(*t*) plots along with snapshots of water spreading at 60 °C. (**a**) Plot of the spreading distance as a function of time between 0 and 120 ms. (**b**) Plot of the velocity as a function of time between 0 and 120 ms. (**c**) Plot of the velocity as a function of time between 0 and 12 ms. (**d**) Plot of the velocity as a function of time between 0 and 70 ms. (**e**) Detailed plot of the spreading distance as a function of time between 0 and 70 ms. (**f**) Plot of the spreading distance as a function of *t*^1/2^.

**Figure 6:**
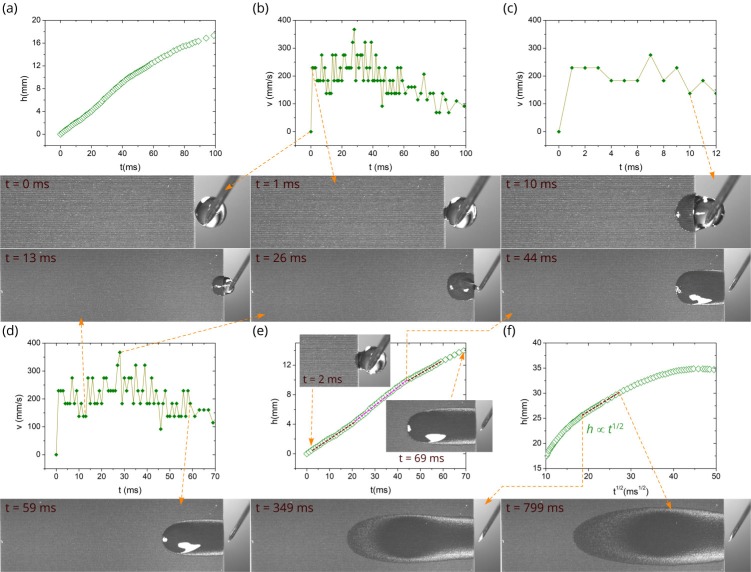
Detailed *h*(*t*) and *v*(*t*) plots along with snapshots of water spreading at 80 °C. (**a**) Plot of the spreading distance as a function of time between 0 and 100 ms. (**b**) Plot of the velocity as a function of time between 0 and 100 ms. (**c**) Plot of the velocity as a function of time between 0 and 12 ms. (**d**) Plot of velocity as a function of time between 0 and 70 ms. (**e**) Detailed plot of the spreading distance as a function of time between 0 and 70 ms. (**f**) Plot of the spreading distance as a function of *t*^1/2^.

Videos S1–S3 in Supplementary Materials show the capillary spreading of water on the sample both in real time and slow motion modes at the studied temperatures. The plots of the spreading distance *h* as a function of time *t* at various temperatures *T* of the sample are shown in Figure 3a,b. It is seen that the created wicking material retains its capillary performance in the studied temperature range, clearly indicating its suitability for cooling and other applications. Initially, capillary spreading is about the same for all temperatures. However, at a certain time (about 50 ms, see Figure 3b), the *h*(*t*) curves begin to divert, exhibiting larger spreading distance, i.e., better capillary action, at 60 and 80 °C as compared with that at 23 °C. As seen from Figure 3a, at 2200 ms the spreading distance is 33, 40.8, and 34.8 mm at 23, 60, and 80 °C, respectively. Also, one can see that the water front reaches the end of the laser-treated area (*h* = 45 mm) at 5809 and 3543 ms for *T* = 23 and 60 °C, respectively. These observations demonstrate that the enhanced wicking functionality at elevated temperatures is significant and holds for relatively longer time. However, with time the evaporation effect comes into play and causes the wetted area to recede, as seen from the behavior of *h*(*t*) at 80 °C. Both spreading and receding regimes of water behavior at 80 °C are shown in Figure 3a, where we can see that the change from the spreading regime to receding one occurs at 2000 ms and the water film completely vaporizes at about *t* = 5000 ms. Figure 3b shows that temperature effects on capillary spreading become noticeable at about 50 ms, shortly after the initiation of capillary flow. The plots of spreading velocity as a function of time at various temperatures presented in Figure 3c,d demonstrate that the qualitative behaviors of *v*(*t*) dependencies in the spreading regime are rather similar at different temperatures. It is seen that initially the spreading velocity jumps up to about 370 mm/s and then quickly drops to a value of about 35 mm/s followed by a slow decrease after about 400 ms. The data presented in Figure 3 show that the most dramatic changes in the spreading distance and spreading velocity occur in the initial stages of capillary flow. To understand the temperature effects on the capillary flow regimes, we perform a detailed analysis of both spreading distance *h*(*t*) and velocity *v*(*t*) dependencies. In this analysis, we mainly focus on the liquid flow at *t* < 200 ms when the most dramatic changes in both *h*(*t*) and *v*(*t*) are observed. The results of the detailed analysis are presented in Figure 4, Figure 5 and Figure 6 at 23, 60, and 80 °C, respectively.

Figure 4 shows the detailed *h*(*t*) and *v*(*t*) plots along with snapshots of water spreading for the room temperature (the corresponding Video S1 of capillary flow can be found in Supplementary Materials). As seen in Figure 4a, the spreading distance achieves a large value (about 15.4 mm) at 100 ms after the contact of the water drop with the capillary structure, indicating a high spreading velocity. The *v*(*t*) plots derived from the *h*(*t*) data are shown in Figure 4b,c, where we can see that initially the velocity jumps to a value of 275 mm/s at *t* = 2 ms and then fluctuates relative to a value of 230 mm/s, taking only three discrete values of 180, 230, and 275 mm/s. At *t* = 20 ms the velocity increases further (see Figure 4b), taking discrete values fluctuating relative to a value of 320 mm/s and achieving a maximum value of 370 mm/s at *t* = 26 ms. In our study, the velocity is derived from the *h*(*t*) data as a numerical derivative Δ*h*/Δ*t*, where Δ*h* is the difference of spreading distance between two consecutive video frames and Δ*t* = 10^−3^ s (in our video recording, the speed is 10^3^ fps and exposure time is 40 μs). The spatial resolution of video recording is about 50 μm. This gives an estimation of the velocity derivation uncertainty of about 50 mm/s. Thus, the observed velocity fluctuations are within this uncertainty and can be attributed to it. The snapshots of the drop dynamics in Figure 4 show that the relocation of the drop from the needle to the sample completes at *t* = 26 ms. The spreading velocity begins clearly to decrease at *t* = 31 ms, undergoing a quick drop in a time domain up to 160 ms (see Figure 3c,d). As seen in Figure 3c, the velocity decrease becomes slow at about *t* > 350 ms when the Washburn regime takes place. 

In general, the liquid flow in a capillary medium at room temperature may follow *h* ∝ *t*^2^ [52,53,54], *h* ∝ *t* [39,52,53,55,56], *h* ∝ *t*^1/2^ [57,58,59,60], *h* ∝ *t*^1/3^ [61], and other dynamics, including transition regimes and final stage [54,55,61]. The *h* ∝ *t*^2^ and *h* ∝ *t* dynamics are referred to as early stages of capillary flow that precede classic Washburn’s *h* ∝ *t*^1/2^ regime [57], in which the capillary force is balanced by viscous drag. The Washburn regime is observed in a large variety of capillary media and believed to be the universal capillary flow law. Therefore, it is important in capillary flow analysis to identify it first. The *h*(*t*^1/2^) plot in Figure 4f shows that Washburn’s flow in our capillary structure begins at 359 ms (*h* = 20.9 mm) and continues up to 5809 ms when the water front reaches the end of the laser-treated area (*h* = 45 mm). Thus, the length of our sample is not sufficient for tracking the Washburn flow up to its end. Washburn’s *h*(*t*^1/2^) flow regime in our sample is indicated in *h*(*t*) and *v*(*t*) plots shown in Figure 3a,c, where we can see that the capillary flow velocity in the early regimes is about by an order of magnitude higher than in Washburn’s stage. This observation shows us the importance of the early stages in solving the problem of quick remediation of dry-out spots, necessitating solid understanding of the early stages. However, in contrast to the Washburn dynamics, even a basic understanding of these regimes is still lacking, especially of the *h* ∝ *t*^2^ one. In the *h* ∝ *t*^2^ regime, the liquid undergoes initial acceleration [52], resulting in a velocity jump. As seen in Figure 4c, in our experiment, the velocity increases in a time domain 0 < *t* < 2 ms. The *h* ∝ *t*^2^ scaling law assumes that the acceleration is caused only by the capillary force generated by a wicking medium and fluid acceleration is constant. In our experiment, in addition to the capillary pressure induced by the surface structure, there is also Laplace pressure produced by the pendant drop curvature that changes with time because of reducing the drop volume [62,63], resulting in a more complicated acceleration process with more than one acceleration stages as observed here. The *h* ∝ *t* flow regime [39,52,53,55,64], referred to as inertial or inviscid, comes after the *h* ∝ *t*^2^ one. In this regime, the capillary force is balanced by the inertial one and the velocity is constant. The previous studies on the *h* ∝ *t* regime relate to the simplest capillary geometries. Our capillary structure has a very high geometrical complexity because of both nano- and micro-capillary geometries, resulting in a more complicated capillary flow, as seen from the data in Figure 4. In our experiment, the *h* ∝ *t* regime can be associated with a time domain between 3 and 20 ms (see Figure 4d,e), after which the second acceleration of liquid flow occurs, where the velocity jumps up to 330–380 mm/s and the water flow retains this range of increased speeds until *t* = 31 ms (Figure 4d). The snapshots at 20, 26, and 31 ms in Figure 4 indicate that the water flow between 20 and 31 ms is driven by the capillary pressure of surface structure, Laplace pressure from the curvature of the drop located on the sample, gravitational force of this drop, and Laplace pressure from the curvature of the drop remaining between the sample edge and needle. At *t* = 31 ms the drop relocation from the needle to sample finishes and the velocity decreases with significant fluctuations. Figure 4d,e and associated snapshots at *t* = 31 and 45 ms show the initial velocity decrease between 31 and 45 ms in detail. Subsequently, the velocity drop continues, taking fluctuating values. Previously, Lade et al. have observed a similar velocity behavior for spreading of aqueous glycerol solution in a single open microchannel with surface roughness and attributed the velocity variations to pinning/depinning effects caused by microgroove roughness [65]. The pinning/depinning effects on velocity variations should also be present in our capillary geometry because of the structured surface of microgrooves. However, they are obscured by the velocity fluctuations coming from the velocity derivation uncertainty. To gain clearer insight into the velocity fluctuations, a study on the liquid flow using higher both temporal and spatial resolutions than used here (1 ms and 50 μm, respectively) is needed. The velocity fluctuations attenuate with time and become small when the liquid flow reaches Washburn’s regime. Figure 3c,d shows that the rapid drop velocity occurs in an exponential manner in a time domain between about 60–300 ms prior to the Washburn regime. Therefore, this time domain can be associated with the visco-inertial flow regime [55]. In summary, our results obtained at room temperature show that the sequence of flow regimes in the here studied hierarchical capillary structure is much more complicated than in simple capillary geometries like tubes and open microgrooves with smooth surfaces. The capillary flow velocity undergoes significant fluctuations prior to the Washburn’s regime, making the identification of the sequence of early flow regimes more difficult.

As we mentioned above, the temperature effect on the spreading distance comes into play at about 50 ms (see Figure 3b). Figure 5 and Figure 6 demonstrate a more detailed flow dynamics at 60 and 80 °C along with snapshots at characteristic times of liquid flow. One can see that qualitatively both *h*(*t*) and *v*(*t*) dependencies before the Washburn regime are actually similar to those at 23 °C. Figure 5f and Figure 6f show that the Washburn regime also exists at 60 and 80 °C, although its lifetime is shorter. As seen, the Washburn regime timescale is 327–1377 ms and 349–799 ms at 60 and 80 °C, respectively. The initial flow stages (inertial and visco-inertial) in a capillary tube have been discussed in detail by Fries and Dreyer [55]. In the purely inertial time stage, the analytical equation for capillary flow is given by:

(1) $$h=t\sqrt{\frac{2\sigma \cos\theta }{R\rho }}$$

where *σ* is the surface tension, *θ* is the contact angle, *ρ* is the fluid density, and *R* is the capillary radius. In this regime, the viscous drag is negligible. At a certain time, the viscous force comes into play, and the liquid flow is governed by both inertial and viscous forces. In this regime, referred to as the visco-inertial, the capillary flow is given by [55]:

(2) $$h^{2}=\frac{2b}{a}\left[t-\frac{1}{a}\left(1-\exp\left(-at\right)\right)\right]$$

with

(3) $$a=\frac{8\mu }{R^{2}\rho }$$

and

(4) $$b=\frac{2\sigma \cos\theta }{R\rho }$$

where *μ* is the viscosity of the fluid. With time the effect of inertia becomes negligible and purely viscous flow stage (Washburn’s regime) begins [55], where

(5) $$h^{2}=\frac{\sigma R\cos\theta }{2\mu }t$$

Analysis of Equations (1)–(5) show that the temperature-dependent parameters in these equations are the contact angle, surface tension, viscosity, and density of the liquid. The contact angle of de-ionized water on silicon decreases with increasing temperature [66], thus being a factor that promotes the capillary flow. The table data of the water surface tension as a function of temperature published in [67] show that the surface tension decreases with temperature (*σ* = 71.99, 66.24, and 62.67 mN⋅m^−1^ at 25, 60, and 80 °C, respectively), thus enhancing the capillary action at higher temperatures in all flow regimes given by Equations (1)–(5). The viscosity of water significantly reduces as the temperature rises [68,69], for example its value is *μ* = 9 × 10^−4^ Pa⋅s at 25 °C, whereas *μ* = 2.6 × 10^−4^ Pa⋅s at 80 °C [69]. As seen from Equations (2)–(5), the viscosity plays a role in visco-inertial and viscous (Washburn’s) flow regimes and its decrease enhances the liquid flow in these regimes. It is known that water has maximum density of 1000 kg/m^3^ at 4 °C, and increasing the temperature above 4 °C causes the water density to decrease to 996.999, 983.154, and 971.761 kg/m^3^ at 25, 60, and 80 °C, respectively [68], thus improving the capillary action. The temperature effect of the water density may play a role in the inertial and visco-inertial regimes. Thus, the temperature effects of *θ*, *σ*, *μ*, and *ρ* are favorable for enhancing the capillary action with increasing temperature. As discussed above, the temperature effects in our capillary structure begin to play at *t* ≈ 50 ms. This time delay can be explained as follows. In our study, the pendant water drop on the syringe needle is at room temperature before the contact with the silicon sample. Therefore, a fraction of water that is in contact with the capillary structure must be first heated to activate the temperature effects on the capillary performance. It is known that the thermal diffusion length *L*_D_ is given by:

(6) $$L_{D}\approx \sqrt{Dt}$$

where *D* is the thermal diffusivity of a material. Using *D* = 0.0014 cm^2^·s^−1^ for water at room temperature [70] and assuming that the water film thickness in the capillary structure is about the depth of the microgrooves (51 µm), we can estimate a characteristic timescale *t*_d_ of heating the water film using Equation (6) at *L*_D_ = 51 µm. This estimation gives us *t*_d_ ≈ *L^2^/D* ≈ 19 ms, which is in a reasonable agreement with the observed *t* ≈ 50 ms. The smaller value of the estimated *t*_d_ can be explained by small temperature effects of *σ*, *θ*, and *ρ* in the inertial regime due to square root function in the Equation (1).

At a certain time of capillary flow, evaporation begins to affect the spreading dynamics, causing the flow velocity to decrease that eventually results in a complete stop of liquid spreading. Two characteristic times are important in analyzing the effect of evaporation on spreading dynamics. The first one is the time when evaporation begins to affect the spreading dynamics; and the second one is the time when evaporation stops the propagation of the capillary flow front and causes the spreading regime to change into a receding one, where the drying front propagates backward toward the water reservoir [50]. Figure 3a shows that at *T* = 80 °C the complete stop of liquid spreading and the change into the receding regime occur at about 2000 ms. At this time the capillary spreading distance reaches its maximum value of 35 mm. The onset of a noticeable evaporation effect on the spreading distance can be found from a comparison of *h*(*t*) dependences in Figure 3a, where it is seen that the initially increasing gap between *h*(*t*) at 23 °C and *h*(*t*) at 60 or 80 °C begins to decrease at about 1700 ms at 60 °C and 900 ms at 80 °C, indicating the onset of noticeable evaporation effect. It is worth noting that the Washburn regime begins at 359, 327, and 349 ms at 23, 60, and 80 °C, respectively, i.e., at about the same time independently of the temperature, whereas its end time is shorter at higher temperatures (>5700, 1377, and 799 ms at 23, 60, and 80 °C, respectively) as seen from Figure 4f, Figure 5f and Figure 6f. These end times of the Washburn regime at 60 and 80 °C are close to the characteristic onset time of noticeable evaporation effect found above, indicating a dominant role of evaporation on shortening the Washburn regime lifetime with increasing temperature. Finally, we note that our experiments are conducted at the fixed humidity. However, evaporation and consequently capillary spreading depend on humidity of the ambient gas medium. An insight into the effect of humidity on capillary spreading has been recently gained in [50].

## 4. Conclusions and Outlook

This work shows that the hierarchical capillary surface structure produced on silicon using the direct femtosecond laser nano/microstructuring technique exhibits excellent capillary performance in the temperature range between 23 and 80 °C. The created silicon material demonstrates very high water flow velocities with a maximum value of 370 mm/s in a time domain *t* < 50 ms, thus providing fast liquid transport for a long distance (up to 10 mm) even during early capillary flow stages preceding the onset of the visco-inertial regime. The classic Washburn’s regime exists at all studied temperatures, giving the evidence of its universality at high temperatures as well. Its onset time insignificantly depends on the temperature; however, its duration considerably shortens with increasing temperature due to evaporation. The finding of great importance is the enhancement of capillary functionality as the temperature rises. This finding along with the high speed of liquid transport provides a way for solving the problem of dry-out spots in cooling devices of 5G electronics. Furthermore, the results of this work are of great significance for engineering high-temperature superwicking materials for heat/mass exchangers needed in such technologically important areas as M-cycle technologies for increasing efficiency of the power generation in gas turbines and reducing fuel consumption in internal combustion engines along with decreasing NO*_x_* emission [41,42,43].

## Supplementary Materials

The following are available online at https://zenodo.org/record/3701547, Video S1: Capillary flow of water on the laser-treated silicon surface at room temperature. Video S2: Capillary flow of water on the laser-treated silicon surface at 60 °C. Video S3: Capillary flow of water on the laser-treated silicon surface at 80 °C.
